# Influence of Vertical Facial Growth Pattern on Herbst Appliance Effects in Prepubertal Patients: A Retrospective Controlled Study

**DOI:** 10.1155/2020/1018793

**Published:** 2020-01-11

**Authors:** Maria Rita Giuca, Marco Pasini, Sara Drago, Leonardo Del Corso, Arianna Vanni, Elisabetta Carli, Antonio Manni

**Affiliations:** ^1^Department of Surgical, Medical, Molecular Pathology and Critical Area, Unit of Pediatric Dentistry, University of Pisa, Pisa, Italy; ^2^Department of Orthodontics, School of Dentistry, University of Genova, Genova, Italy; ^3^Private Practice, Livorno, Italy; ^4^Private Practice, Pisa, Italy; ^5^Private Practice, Racale, Lecce, Italy

## Abstract

**Introduction:**

The Herbst device is widely used for correction of class II malocclusions; however, most of the researches carried out on the Herbst appliance in literature do not take into account patients with a different mandibular divergence. The aim of this study was to investigate the effects of Herbst on dental and skeletal structures and to evaluate possible influence of vertical facial growth patterns.

**Methods:**

A retrospective study was conducted on lateral cephalograms of 75 growing patients (mean age: 9.9 ± 1.9 years) with class II malocclusion treated with Herbst. Subjects were divided into 3 groups using the mandibular divergence index (SN and GoMe angle). Cephalometric parameters were evaluated using the modified SO (sagittal occlusion) Pancherz's analysis. A statistical analysis was conducted to evaluate differences among groups using ANOVA.

**Results:**

Our study showed differences in response to treatment depending on patient's facial vertical growth pattern. Cranial base angle and mandibular rotation were significantly different (*p* < 0.05) between hypodivergent patients and normodivergent patients and between hypodivergent and hyperdivergent subjects.

**Conclusion:**

Hypodivergent patients increased their mandibular divergence during treatment to a greater extent than normodivergents; moreover, hyperdivergent patients exhibited a decreased mandibular divergence at the end of the treatment.

## 1. Introduction

Bilateral class II malocclusion represents one of the main orthodontic problems affecting the world population, and it has been observed that this condition affects 27.2% of English adolescents [1], 36.3% of Italian adolescents [2], about 15% of the total United States population [3], and 27.0% of Chinese children [4].

This sagittal malocclusion can be skeletal, dental, or combined. In particular, in the great majority of cases (about 75%), the skeletal component is affected [5].

The Herbst device is one of the most common appliances for the treatment of skeletal and dental class II, consisting of a piston and a tube anchored to orthodontic bands (or to splints or to cobalt/chrome fusions), which keeps the jaw in a protracted position 24 hours a day [6] through a bilateral telescopic mechanism.

The advantages include the following: high treatment speed (average treatment time 6–8 months), reduced request for patient's compliance, and effectiveness both on the dental and skeletal component [7].

The effects on the dental component include a distalisation of the upper dental arch and a mesialisation of the lower dental arch [8], while the effects on the skeletal component include a decreased growth of the maxilla [9] and a stimulation of the mandibular growth with an increase in the average length at the end of treatment greater than 2-3 mm [10]. The mechanism permits vertical opening movements and effect on the vertical tooth position [11], and the skeletal effect is most pronounced during puberty rather than before [12].

The main disadvantage of the Herbst consists in a proclination of lower incisors due to anchorage loss in different amounts relative to the type of Herbst used [13]; various modifications of the original orthodontic appliance have been proposed, but none has been able to completely prevent proclination of mandibular incisors [13–15]. Most of the studies carried out on the Herbst appliance do not take into account patients with a different mandibular divergence [15], which affects chin position [16], the direction of the condylar growth [17], and the shape of the jaw [18].

Variation in the mandibular divergence with other orthodontic appliances for class II malocclusions has been investigated in a recent systematic review [19].

The aim of this study was to investigate the effect of the Herbst appliance on dental and skeletal levels and to evaluate the existing differences between patients with different vertical growth patterns.

## 2. Materials and Methods

A retrospective study was conducted on lateral cephalograms of consecutive patients previously treated in a private office (Lecce, Italy) within the past 5 years: from January 2014 to January 2019.

Sample size calculation was performed; estimate of standard deviation was based on data obtained from other 10 subjects who were followed in a preliminary study, considering mandibular divergence as the primary outcome. In order to compare two means with a power of 80%, a size of the test of 5%, a standard deviation of 1.5, and a difference of 1.2, the sample size required 25 patients in each group.

A total of 75 lateral cephalograms of patients with a skeletal class II and treated with Herbst appliance (35 males and 40 females; average age at the start of treatment 9.9 ± 1.9 years; average Herbst treatment duration 9.7 ± 1.6 months) were included in this study (test group).

The test group was compared with a control group of 75 untreated subjects, obtained from the University of Michigan Growth Study Center, the Bolton-Brush Growth study center, the University of Toronto Burlington Growth Study, the University of Oklahoma Denver Growth Study, the Oregon Growth Study, the Iowa Facial Growth Study, and the UOP Mathews Growth Study, matched for similar vertical relationships, sex, and skeletal age.

All procedures were conducted according to the principles expressed in the Declaration of Helsinki (1964), and a written consent (signed by parents or legal guardians) to participate in the study was obtained at the beginning of the orthodontic treatment.

The inclusion criteria were as follows: lateral cephalograms taken before and after Herbst treatment, presence of a permanent dentition or late mixed dentition, presence of bilateral angle class II division 1 malocclusion, and presence of mandibular deficiency and normal upper jaw. Exclusion criteria were as follows: presence of serious skeletal malformations, patients with systemic disease, patients undergoing a drug therapy that may cause skeletal abnormalities, and patients with agenesis and/or premature loss of permanent teeth. Lateral cephalograms were divided into 3 groups using the mandibular divergence index, measured on lateral cephalograms at the beginning of the treatment: angle between the straight lines SN (Sella-Nasion) and GoMe (Gonion-Menton).

All subjects with SN^GoMe values less than or equal to 26.5° were considered as belonging to the hypodivergent group, all subjects with SN^GoMe values between 26.5° and 36.5° were considered as belonging to the normodivergent group, and all subjects with SN^GoMe values greater than or equal to 36.5° were considered as belonging to the hyperdivergent group.

The test group consisted of three different subgroups: group 1 included 25 hypodivergent subjects (12 males and 13 females) with an average age at the start of treatment of 10.6 ± 2.0 years and a mean duration of Herbst treatment of 9.6 ± 1.9 months.

Group 2 included 25 normodivergent subjects (11 males and 14 females) with an average age at the beginning of treatment of 9.8 ± 1.9 years and a mean duration of orthodontic treatment of 9.5 ± 1.7 months.

Group 3 included 25 hyperdivergent subjects (12 males and 13 females) with a mean age at the start of treatment of 9.4 ± 1.8 years and an average duration of treatment of 9.9 ± 1.3 months. Each subgroup was compared with three different control groups of 25 lateral cephalometrics, matched with the test subgroup for similar SN^GoMe value, sex, and skeletal age that was assessed with cervical vertebral maturation staging [20].

### 2.1. Cephalometric Parameters

The investigation of the Herbst appliance effects at the dental and skeletal levels was performed on lateral cephalograms using the modified SO (sagittal occlusion) Pancherz's cephalometric analysis.

**Figure 1:**
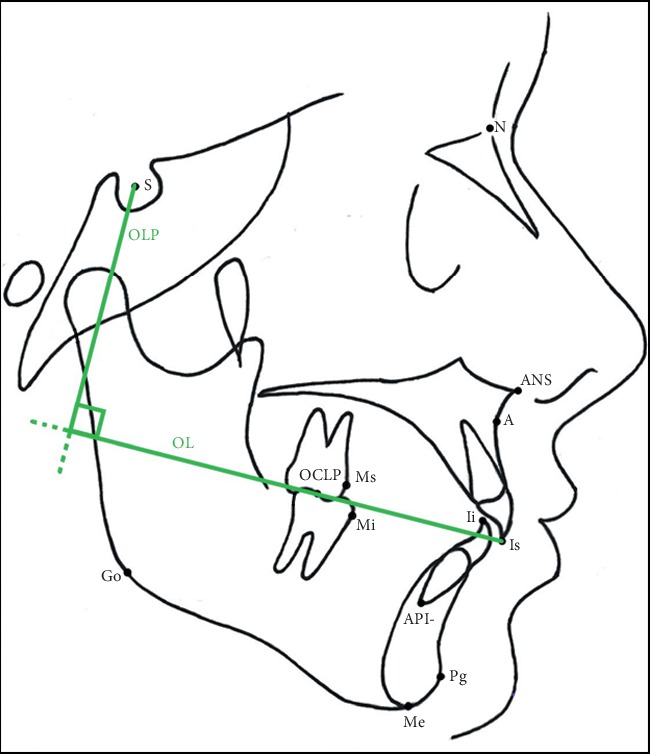
Modified SO Pancherz analysis. Reference points and lines: Sella (S), Nasion (N), subnasal (A), pogonion (Pg), Gonion (Go), Menton (Me), articular (Ar), anterior nasal spine (ANS), maxillary incisal (Is), mandibular incisal (Ii), lower incisal apex (API), posterior occlusal (OCLP), maxillary molar (Ms), mandibular molar (Mi), occlusal line (OL), and occlusal line perpendicular (OLP).

This analysis was carried out by transferring the lines occlusal line (OL) and occlusal perpendicular line (OLp) through the Sella from pretreatment lateral cephalogram to the posttreatment lateral cephalogram by superimposing skeletal stable structures of the anterior cranial base. Modified SO Pancherz's cephalometric analysis included the following parameters, that are not considered in traditional SO Pancherz analysis: skeletal divergence, skeletal class, and lower incisor inclination (Figure 1).

Cephalograms were performed with teeth in centric occlusion, with relaxed lips and head oriented parallel to the floor according to the Frankfurt plane.

For each patient of the test group, two lateral cephalograms were included: pretreatment (T1) and posttreatment (T2).

Cephalometric analysis was performed by a single operator using Delta-Dent® software (Orthopiù SRL).

### 2.2. Statistical Analysis

All linear and angular measurements were approximated to the nearest 0.1 mm and 0.1°, respectively. Dahlberg's formula was adopted after measuring each lateral cephalogram twice, with 14 days between each measurement; the method error was less than 0.5 mm and 1 degree (intraoperator reliability).

A blinded statistical analysis was performed. Data were checked for normality using the Shapiro–Wilk test. Continuous variables are given as means and standard deviations (SD), whereas categorical variables were given as number and/or percentage of subjects. The thirteen cephalometric parameters were considered as primary outcome measurements. Outcome baseline differences among treatment groups were tested by one-way ANOVA. In order to investigate the associations of the outcome parameters with divergence groups, the one-way ANOVA was performed again on the differences after-before for each group. A paired *t*-test was performed to observe intragroup differences. Subsequently, an independent samples *t*-test was adopted to evaluate the differences between each group and the controls.

The estimated *p* values were adjusted for multiple comparisons by the Bonferroni correction method, and when the adjusted *p* value was less than 0.05, the differences were selected as significant. Data were acquired and analysed in R v3.4.4 software environment.

## 3. Results

No significant differences between groups were detected at baseline except for SN^GoMe, lower incisor axis inclination, AN^NPg, and skeletal discrepancy (*p* < 0.001, Table 1).

Table 2 shows for each treatment group any difference over time in all measurements; the ANOVA assessed a significant difference over time among groups for the following parameters: Ii-Olp and SN^GoMe.

Significant intragroup variations from T1 to T2 in the total sample and in the three test subgroups are summarized in Table 3.

Herbst therapy has determined in the total sample a slight retreat of the upper maxilla, but no significant difference was observed at the end of the treatment. On the contrary, a significant advancement of the lower jaw with a reduction of skeletal discrepancy and improvement of ANPg angle was found after the Herbst treatment (*p* < 0.05).

Moreover, orthodontic treatment resulted in a slight retreat of the upper central incisor even if the difference was not significant (*p* > 0.05.05), a marked advancement of the lower central incisor (*p* < 0.05), and a marked reduction of overjet and molar relation (*p* < 0.05).

In the total sample, a loss of dental anchorage with an increased lower incisor inclination at the end of the treatment (*p* < 0.05) and a mean increase in cranial base-mandible angle (SN/GoMe) was observed.

Cephalometric changes (T2-T1) in the three subgroups are reported in Table 4 (hypodivergents vs. controls), Table 5 (normodivergent vs. controls), and Table 6 (hyperdivergent vs. controls).

Hypodivergent patients showed an increased mandibular divergence at the end of the therapy in comparison to the control group (*p* < 0.05), normodivergent subjects did no show significant changes in divergence in comparison to the controls (*p* > 0.05), and hyperdivergents showed a decrease in SN/GoMe angle in comparison to the control group (*p* < 0.05).

## 4. Discussion

Based on the results obtained in this study, it is possible to notice that the Herbst treatment was effective for the resolution of class II malocclusion in all groups.

In fact, correction of sagittal dental class was obtained in all patients treated, with a decrease in overjet, skeletal class angle, skeletal discrepancy, and molar relation.

These results were obtained in all patients through a distalisation of the upper arch and a mesialisation of the lower arch, and these results are consistent with those of previous studies [8, 10, 17, 21–23]. A slight high-pull headgear effect on the maxillary complex was found in the total sample, while a significant advancement of the mandible was observed (hypodivergents exhibited a slight lower mandibular advancement in comparison to normodivergent and hyperdivergent groups), and these results are in accordance with those of previous studies; Pancherz and Anehus-Pancherz found that the sagittal maxillary jaw base position seemed unaffected by therapy [24]. An increase in the inclination of the lower incisor, with respect to the mandibular base, was recorded in all groups, and a greater mandibular incisor anchorage loss was observed in the hyperdivergent group, while hypodivergent exhibited the lower mandibular incisor anchorage loss. However, no significant differences in mandibular advancement were found among groups.

Normodivergents did not show changes in divergence; hypodivergent patients slightly increased their mandibular divergence during orthodontic treatment, while hyperdivergent patients showed a slight decrease in the mandibular divergence.

In literature, a significant alteration was found in mandibular divergence at the end of the Herbst treatment in a limited number of studies [25–27], while other previous studies showed a significant change of SN/GoMe at the end of orthodontic therapy [28].

It was recorded that, after Herbst treatment, the upper molars moved mesially, the occlusal plane slightly closed, and the palatal plane tipped downward [24].

Ruf and Pancherz stated that the mandibular plane angle was slightly affected by Herbst appliance treatment, and at the end of the orthodontic therapy, a continuous decrease in the mandibular plane angle was found [25].

In a previous study, a significant difference of cranial base-mandibular angle was found between hypodivergent, normodivergent, and hyperdivergent patients [28], and the results showed that hypodivergent subjects tend to decrease this angle, while hyperdivergents tend to increase it. In fact, these authors observed that hypo- and hyperdivergent patients benefit from Herbst's headgear effect in the upper maxilla, while hyperdivergent patients exhibited a deleterious backward mandibular rotation. A possible explanation could be that cantilever Herbst appliance with full-coverage stainless steel crowns on the upper and lower first molars was used by Rogers et al. [28], while in the present study, a total acrylic splint extending from the first lower molar to the first contralateral molar was used to reinforce the anchorage. Furthermore, another possible explanation for the rotational differences between subjects with different vertical growth patterns could be the orofacial musculature function as patients with weak jaw musculature could exhibit a backward mandibular rotation.

Further studies conducted on a larger number of lateral cephalograms will be necessary to confirm the results of the present study.

## 5. Conclusion

Our study showed differences in response to treatment with the Herbst appliance depending on patient's vertical growth pattern. Particularly, the changes in Ii/Olp over time were significantly different among groups (*p* < 0.001).

Moreover, the results exhibited that hypodivergent patients increased their mandibular divergence during treatment. Normodivergent patients showed very slight differences in mandibular divergence with no significant difference, while hyperdivergent patients exhibited a mandibular divergence decrease at the end of the Herbst treatment, and the difference among groups was significant (*p* < 0.05).

## Figures and Tables

**Table 1:** Baseline characteristics in whole population (*N*=75).

Outcome variables	Total mean (SD)	Group			*p* value	Pairwise comparisons
		Hypodivergent mean (SD)	Normodivergent mean (SD)	Hyperdivergent mean (SD)		
A/Olp	69.91 (4.47)	69.68 (5.25)	70.38 (4.46)	69.67 (3.71)	0.817	
Pg/Olp	69.97 (5.87)	70.89 (6.38)	70.13 (6.19)	68.88 (5.02)	0.481	
Is/Olp	77.60 (4.86)	76.94 (6.06)	77.79 (4.43)	78.08 (3.95)	0.693	
Ii/Olp	70.29 (5.42)	70.32 (6.23)	70.19 (5.27)	70.36 (4.90)	0.993	
Ms/Olp	36.53 (4.54)	36.78 (5.14)	36.68 (4.79)	36.13 (3.74)	0.864	
Mi/Olp	34.84 (5.39)	34.84 (6.35)	34.78 (5.12)	34.92 (4.80)	0.996	
SN^GoMe	32.67 (5.97)	25.68 (2.31)	33.09 (2.02)	39.24 (2.15)	<0.001	Hypo vs. hyper: <0.001
						Hypo vs. normo: <0.001
						Hyper vs. normo: <0.001
Lower incisor axis inclination	100.26 (6.57)	104.62 (6.56)	99.54 (4.46)	96.62 (6.00)	<0.001	Hypo vs. hyper: <0.001
						Hypo vs. normo: <0.001
						Hyper vs. normo: 0.2315
AN^NPg	4.78 (2.30)	3.35 (2.01)	5.40 (1.93)	5.59 (2.32)	<0.001	Hypo vs. hyper: <0.001
						Hypo vs. normo: <0.001
						Hyper vs. normo: 1.00
Skeletal discrepancy	−0.07 (2.68)	−1.24 (2.67)	0.24 (2.78)	0.79 (2.21)	0.019	Hypo vs. hyper: 0.02
						Hypo vs. normo: 0.13
						Hyper vs. normo: 1.00
Overjet	7.32 (2.47)	6.70 (2.51)	7.55 (2.64)	7.72 (2.23)	0.298	
Molar relation	1.69 (1.92)	1.96 (2.14)	1.90 (1.65)	1.21 (1.93)	0.312	

Results are expressed as mean (standard deviation); *p* value = one-way ANOVA; *p* value, pairwise comparisons: *p* values adjusted by using the Bonferroni method.

**Table 2:** One-way ANOVA results to evidence any difference over time between groups.

Outcome variables	Total mean (SD)	Group			*p* value	Pairwise comparisons
		Hypodivergent mean (SD)	Normodivergent mean (SD)	Hyperdivergent mean (SD)		
A/Olp	0.18 (1.58)	0.35 (1.92)	−0.23 (1.32)	0.40 (1.42)	0.296	
Pg/Olp	2.33 (2.19)	2.11 (2.31)	2.41 (2.02)	2.46 (2.31)	0.837	
Is/Olp	−0.43 (2.04)	0.17 (2.42)	−0.64 (1.80)	−0.82 (1.78)	0.190	
Ii/Olp	0.36 (3.57)	−2.72 (2.74)	4.00 (1.91)	−0.19 (2.03)	<0.001	Hypo vs. hyper: <0.001
						Hypo vs. normo: <0.001
						Hyper vs. normo: <0.001
Ms/Olp	−1.36 (2.20)	−1.61 (2.41)	−1.44 (2.53)	−1.03 (1.61)	0.640	
Mi/Olp	4.07 (2.52)	3.56 (2.91)	4.36 (2.42)	4.28 (2.20)	0.473	
SN^GoMe	0.28 (2.77)	1.66 (2.53)	−0.01 (2.33)	−0.81 (2.91)	0.004	Hypo vs. hyper: 0.004
						Hypo vs. normo: 0.077
						Hyper vs. normo: 0.842
Lower incisor axis inclination	5.58 (4.77)	4.51 (3.84)	5.47 (5.52)	6.77 (4.72)	0.246	
AN^NPg	−1.49 (1.72)	−1.20 (1.27)	−2.05 (2.16)	−1.24 (1.52)	0.142	
Skeletal discrepancy	−2.52 (2.28)	−3.08 (2.66)	−2.43 (2.09)	−2.06 (1.99)	0.281	
Overjet	−4.38 (2.21)	−3.62 (2.07)	−4.64 (2.19)	−4.87 (2.26)	0.102	
Molar relation	−5.02 (5.54)	−5.53 (3.22)	−4.21 (8.76)	−5.31 (2.50)	0.672	

Pairwise comparisons: *p* values adjusted by using Bonferroni method. In the first column, differences after-before for each group are given.

**Table 3:** Intragroup *p* values (test group: T2-T1).

	Total sample (75)	Hypodivergent (25)	Normodivergent (25)	Hyperdivergent (25)
	*p*	*p*	*p*	*p*
A/Olp	0.90	0.81	0.88	0.85
Pg/Olp	0.02^*∗*^	0.50	0.14	0.11
Is/Olp	0.45	0.74	0.76	0.50
Ii/Olp	0.01^*∗*^	0.10	0.01^*∗*^	0.01^*∗*^
Ms/Olp	0.07	0.20	0.30	0.38
Mi/Olp	0.01^*∗*^	0.07	0.01^*∗*^	0.01^*∗*^
SN^GoMe	0.97	0.05	0.93	0.37
Lower incisor axis inclination	0.01^*∗*^	0.04^*∗*^	0.01^*∗*^	0.01^*∗*^
AN^NPg	0.01^*∗*^	0.06	0.01^*∗*^	0.07
Skeletal discrepancy	0.01^*∗*^	0.04^*∗*^	0.01^*∗*^	0.01^*∗*^
Overjet	0.01^*∗*^	0.01^*∗*^	0.01^*∗*^	0.01^*∗*^
Molar relation	0.01^*∗*^	0.01^*∗*^	0.01^*∗*^	0.01^*∗*^

^*∗*^ *p* < 0.05.

**Table 4:** Hypodivergent patients versus controls, mean difference between posttreatment (T2) and pretreatment (T1).

Parameter	Hypodivergent test mean (SD)	Hypodivergent control mean (SD)	*p* value
A/Olp	0.35 ± 1.92	0.1 ± 0.3	0.5
Pg/Olp	2.11 ± 2.31	0.2 ± 0.5	<0.001
Is/Olp	0.17 ± 2.42	0.1 ± 0.4	0.89
Ii/Olp	−2.72 ± 2.74	0.2 ± 0.5	<0.001
Ms/Olp	−1.61 ± 2.41	0.2 ± 0.4	<0.001
Mi/Olp	3.56 ± 2.91	0.2 ± 0.3	<0.001
SN^GoMe	1.66 ± 2.53	−0.5 ± 0.9	<0.001
LII	4.51 ± 3.84	0.1 ± 0.3	<0.001
AN^NPg	−1.20 ± 1.27	0.2 ± 0.3	<0.001
Skeletal discrepancy	−3.08 ± 2.66	0.1 ± 0.2	<0.001
Overjet	−3.62 ± 2.07	0.1 ± 0.3	<0.001
Molar relation	−5.53 ± 3.22	0.1 ± 0.4	<0.001

**Table 5:** Normodivergent patients versus controls, mean difference between posttreatment (T2) and pretreatment (T1).

Parameter	Normodivergent test mean (SD)	Normodivergent control mean (SD)	*p* value
A/Olp	−0.23 ± 1.32	0.2 ± 0.5	0.13
Pg/Olp	2.41 ± 2.02	0.4 ± 0.7	<0.001
Is/Olp	−0.64 ± 1.80	0.3 ± 0.4	0.01
Ii/Olp	4.00 ± 1.91	0.2 ± 0.4	<0.001
Ms/Olp	−1.44 ± 2.53	0.3 ± 0.6	<0.001
Mi/Olp	4.36 ± 2.42	0.2 ± 0.5	<0.001
SN^GoMe	−0.01 ± 2.33	0.2 ± 0.4	0.66
LII	5.47 ± 5.52	0.2 ± 0.2	<0.001
AN^NPg	−2.05 ± 2.16	0.1 ± 0.2	<0.001
Skeletal discrepancy	−2.43 ± 2.09	0.2 ± 0.3	<0.001
Overjet	−4.64 ± 2.19	0.1 ± 0.4	<0.001
Molar relation	−4.21 ± 8.76	0.1 ± 0.3	<0.001

**Table 6:** Hyperdivergent patients versus controls, mean difference between posttreatment (T2) and pretreatment (T1).

Parameter	Hyperdivergent test mean (SD)	Hyperdivergent control mean (SD)	*p* value
A/Olp	0.40 ± 1.42	0.1 ± 0.9	0.38
Pg/Olp	2.46 ± 2.31	−0.2 ± 1	<0.001
Is/Olp	−0.82 ± 1.78	0.1 ± 0.8	0.02
Ii/Olp	−0.19 ± 2.03	0.1 ± 0.9	0.51
Ms/Olp	−1.03 ± 1.61	0.2 ± 0.7	0.001
Mi/Olp	4.28 ± 2.20	0.2 ± 0.8	<0.001
SN^GoMe	−0.81 ± 2.91	0.5 ± 0.8	0.03
LII	6.77 ± 4.72	0 ± 0.1	<0.001
AN^NPg	−1.24 ± 1.52	0.2 ± 0.6	<0.001
Skeletal discrepancy	−2.06 ± 1.99	0.1 ± 0.5	<0.001
Overjet	−4.87 ± 2.26	0.1 ± 0.4	<0.001
Molar relation	−5.31 ± 2.50	0.1 ± 0.3	<0.001

## Data Availability

The data used to support the findings of this study are available from the corresponding author upon request.

## Supplementary Materials

Supplementary Materials Cephalometric parameters and their description.
