# Supplementary material for: Influence of Vertical Facial Growth Pattern on Herbst Appliance Effects in Prepubertal Patients: A Retrospective Controlled Study
Source: Int J Dent. 2020 Jan 11;2020:1018793. doi: 10.1155/2020/1018793 (PMC7201793; doi:10.1155/2020/1018793)
Supplement: Supplementary Materials — Cephalometric parameters and their description. [file 1018793.f1.docx]

Supplementary material Cephalometric parameters and their description.

| Maxillary bone base A/OLP | Distance from OLP line to point A (mm) |
| --- | --- |
| Mandibular bone base Pg/OLP | Distance from OLP line to the point Pg (mm) |
| Upper incisal position Is/OLP | Distance from OLP line to the point Is (mm) |
| Lower incisal position Ii/OLP | Distance from OLP line to the point Ii (mm) |
| Upper molar position Ms/OLP | Distance from OLP line to the point Ms (mm) |
| Lower molar position Mi/OLP | Distance from OLP line to the point Mi (mm) |
| Skeletal discrepancy A/OLP – Pg/OLP | A/OLP minus Pg/OLP (mm) |
| Overjet Is/OLP – Ii/OLP | Is/OLP minus Ii/OLP (mm) |
| Molar relation Ms/OLP – Mi/OLP | Ms/OLP minus Mi/OLP (mm) |
| Skeletal divergence SN/GoMe | Angle between SN and GoMe (degrees) |
| Skeletal class ANPg | Angle between AN and NPg (degrees) |
| Lower incisal inclination (LII)/GoMe | Angle between GoMe and Lower incisal axis (degrees) |
